# Molecular epidemiology of non-falciparum *Plasmodium* infections in three different areas of the Ivory Coast

**DOI:** 10.1186/s12936-023-04639-7

**Published:** 2023-07-19

**Authors:** Assohoun J. S. Miezan, Akpa P. Gnagne, Akoua V. Bedia-Tanoh, Estelle G. M. Kone, Abibatou A. Konate-Toure, Kpongbo E. Angora, Abo H. Bosson-Vanga, Kondo F. Kassi, Pulchérie C. M. Kiki-Barro, Vincent Djohan, Hervé E. I. Menan, William Yavo

**Affiliations:** 1 UFR of Pharmaceutical and Biological Sciences, Department of Parasitology-Mycology, Félix Houphouët-Boigny University, Abidjan, Côte d’Ivoire; 2 National Institute of Public Health, B.P. V47, Abidjan, Côte d’Ivoire

**Keywords:** *Plasmodium*, Nested PCR, Ivory Coast

## Abstract

**Background:**

Malaria is a major public health problem, particularly in the tropical regions of America, Africa and Asia. *Plasmodium falciparum* is not only the most widespread but also the most deadly species. The share of *Plasmodium* infections caused by the other species (*Plasmodium ovale* and *Plasmodium malariae*) is clearly underestimated. The objective of the study was to determine the molecular epidemiology of plasmodial infection due to *P. malariae* and *P. ovale* in Côte d'Ivoire.

**Methods:**

The study was cross-sectional. The study participants were recruited from Abengourou, San Pedro and Grand-Bassam. Sample collection took place from May 2015 to April 2016. Questionnaires were administered and filter paper blood samples were collected for parasite DNA extraction. The molecular analysis was carried out from February to March 2021. A nested PCR was used for species diagnosis. The data was presented in frequencies and proportions.

**Results:**

A total of 360 patients were recruited, including 179 men (49,7%) for 181 women (50,3%). The overall *Plasmodium* positive rate was 72.5% (261/360). The specific index was 77.4% and 1.5% for *P. falciparum* and *P. malariae* in mono-infection, respectively. There was also 15% *P. falciparum* and *P. malariae* co-infection, 3.4% *P. falciparum* and *P. ovale* co-infection and 2.3% *P. falciparum*, *P. malariae* and *P. ovale* triple-infection. Typing of *P. ovale* subspecies showed a significant predominance of *P. ovale curtisi* (81.2% of cases).

**Conclusion:**

*Plasmodium falciparum* remains the most prevalent malaria species in Côte d'Ivoire, but *P. malariae* and *P. ovale* are also endemic mostly in co-infection. Malaria elimination requires a better understanding of the specific epidemiological characteristics of *P. malariae* and *P. ovale* with a particular emphasis on the identification of asymptomatic carriers.

## Background

Malaria is a major public health concern, affecting the tropical regions of America, Africa, Eastern Mediterranean, South East Asia, and Western Pacific. Approximately 241 million cases and 619,000 deaths were reported in 2021 [1]. Of the four species of *Plasmodium* infecting humans, *Plasmodium falciparum* is the deadliest species as well as the most prevalent in Africa, whereas the incidence of *Plasmodium vivax* infection is higher outside Africa [2]. Besides these two main species, *Plasmodium ovale* and *Plasmodium malariae* have long been widespread in tropical Africa and other major malaria-endemic areas worldwide [3].

Previous studies have reported that in the humid forest and savanna areas of West and Central Africa, high prevalence of both these species is common in children, often reaching 15–40% for *P. malariae* and 4–10% for *P. ovale.* In these studies, thin blood smears were carefully examined by trained microscopists because parasitaemia is usually low [4, 5]. In areas with a long rainy season and/or perennial or semi-perennial *Anopheles* breeding sites, most *P. ovale* and *P. malariae* infections are associated with *P. falciparum* [6, 7]. In previous studies conducted in Côte d'Ivoire, most malaria cases were due to *P. falciparum* (95–99%), followed by *P. malariae* (3–4.2%) and *P. ovale* (0.3–0.7%) [8, 9].

These figures reported for *P. malariae* and *P. ovale* appear to be underestimated because of the low parasitaemia during the infections they induce and the presence of mixed infections with the main parasite species [6, 10]. The advent of polymerase chain reaction (PCR)-based molecular diagnostic methods has revolutionised the detection of *Plasmodium* pauci-infections [11]. In addition, molecular genotyping has led to the division of *P. ovale* into two distinct subspecies: *P. ovale curtisi*, the classical type, and *P. ovale wallikeri*, the alternative type [12]. The persistence of asymptomatic parasitaemia in the population could constitute a reservoir and threaten the achievement of malaria elimination [13, 14]. In addition, *P. ovale* may be responsible for distant relapses from dormant (hypnozoite) forms in the liver [15] and may even cause severe clinical disease in naive travellers [16].

The objective of the study was to determine the molecular epidemiology of plasmodial infections caused by *P. malariae* and *P. ovale* in Côte d’Ivoire.

## Methods

### Type of study and study area

This cross-sectional study was performed in three localities, namely Abengourou, San Pedro (both are sentinel sites for monitoring *P. falciparum* chemoresistance in the Côte d’Ivoire), and Grand Bassam (not a sentinel site for monitoring *Plasmodium* chemoresistance; however, the region is a favourable biotope for the development of malaria).

### Sample collection period and study population characteristics

Overall, sample collection took place from May 2015 to April 2016 in the rainy and dry seasons. In Grand-Bassam, blood samples were collected from 13 to 15th May 2015 during the rainy season and from 19th to 21st January 2016 during the dry season. In Abengourou, sampling took place from 17 to 19th November 2015 during the rainy season, then from 1st to 3rd March 2016 in the dry season. Finally, in San Pedro, blood samples were collected from 23rd to 25th February 2016 in the dry season and then from 19 to 21th March 2016 in the rainy season.

The PCR analysis of the collected samples was carried out at the Centre de Recherche et de Lutte contre le Paludisme of the National Institute of Public Health from February to March 2021.

For each site, Dried Blood Spot (DBS) from samples of schoolchildren with or without malaria symptoms were randomly selected.. Each schoolchild was included after written informed consent was obtained from their parent or legal guardian. The children included in the study met the following criteria: (i) regularly enrolled in one of the randomly selected schools; (ii) from 4 to 16 years old, regardless of gender; (iii) living in the study area for at least 2 months; (iv) any patients were included (whether or not they were carriers of a plasmodial infection.

However, children treated with an antimalarial drug in the 7 days prior to sampling, or with clinical signs of severe malaria, were not included.

For each patient, a venipuncture sample was collected. The blood samples were spotted on Wathman filter paper to make a DBS for molecular diagnosis. A questionnaire was administered to each schoolchild or parent to collect sociodemographic and clinical data. Each blood spot was labelled with the identification number of the individual, date, and site of collection. Blood spots were dried, and stored in individual zip bag until DNA extraction was performed.

### DNA extraction

DNA was extracted using the Quick-DNA^™^ Universal Kit (lot No ZRC 206,021; the Epigenetics company; USA) according to the manufacturer's instructions. Briefly, DNA was attached to a Zymo-Spin IIC-XL centrifugation column filter. The cells were then washed thrice; first wash with DNA Pre-Wash Buffer and then two washes with g-DNA Wash Buffer. Finally, pure DNA was eluted from the centrifugation column filter using DNA Elution Buffer.

### Molecular amplification using nested PCR

The nested PCR technique has been used for molecular diagnosis of *Plasmodium* species [17, 18]. The first step of this method is specific to the *Plasmodium* genus (rPLU5/rPLU6). For species typing, the primary PCR products of the positive samples were further subjected to four secondary amplifications with primer pairs specific for the four species that are likely to infect humans; rFAL1/2, rVIV1/2, rMAL1/2, and rOVA1/2 for *P. falciparum*, *P. vivax*, *P. malariae*, and *P. ovale*, respectively. However, this last primer pair has a limitation of preferentially binding or amplifying the *P. ovale curtisi* [18] Positive and negative controls were included for each PCR. Moreover, 1 µL of DNA was used for the first PCR in a 25-µL reaction mixture or “mix”. Furthermore, 1 µL of the product from the first PCR was then used for the second PCR in a 25 µL mix.

After detection, *P. ovale* isolates were typed to determine the subspecies of *P. ovale* using the nested PCR method described by Fuehrer [19], with the primer pairs rOVA1/rOVA2 specific for *P. ovale curtisi* and rOVA1v/rOVA2v specific for *P. ovale wallikeri*.

### Data analysis

The collected data were coded and entered using Epi Data 3.1, Excel 2010, and Word 2010. Statistical analyses were performed using Statistical Package for Social Sciences version 21 (SPSS Inc., Chicago, IL, USA). Descriptive statistics were used to assess the distribution of sociodemographic data and independent variables as well as the frequencies and proportions of malaria-positive samples.

### Ethical considerations

The study protocol was approved by the National Research Ethics Committee under Number 020/MSLS/CNER-dkn on 05 May 2015. The study was conducted in accordance with the text of the Declaration of Helsinki adopted by the 18th World Medical Assembly in 1964 and its amendments, the International Conference on Harmonisation recommendations. The study complied with Good Clinical Practice guidelines and all applicable regulatory requirements for clinical studies.

## Results

DBS from 360 patients were used for molecular analysis. The distribution of samples collected was 89 (25%) in Grand Bassam, 129 (36%) in Abengourou and 142 (39%) in San Pedro.

### Socio-demographic and clinical characteristics of participant with non-falciparum infections

Among the 360 participants, 261 were diagnosed with malaria infection (72.5%). Out of the 261 malaria-infected cases, 59 were non-falciparum malaria infections. The demographic characteristics of the participants with non-falciparum malaria infection showed that 54.2% (27/59) of the participants with non-falciparum malaria were females. The study population was predominantly female, with a sex ratio of 0.84 (27/32). The mean age of the patients was 8.97 years (standard deviation = 2.75), with extremes of 5 and 16 years. The age group of 6–10 years was the most represented, with 64.4% (38/59) of the cases. Most cases were found in the towns of San Pedro and Abengourou with an identical percentage of 44.1% (26/59) for each. Majority of the samples 66.1% (39/59) were collected during the rainy season (Table 1).

**Table 1:** Socio-demographic characteristics of the study population (non-falciparum cases)

	Number (n)	Percentage
Age range (years)		
0–5	4	6.8
6–10	38	64.4
10–16	17	28.8
Sex		
Female	32	54.2
Male	27	45.8
Locality		
Abengourou	26	44.1
Grand-Bassam	7	11.9
San-Pedro	26	44.1
Season		
Rainy	39	66.1
Dry	20	33.9
Total	59	100.0

On clinical examination, only 11.9% (7/59) schoolchildren selected were febrile; the majority of those selected had a normal temporal temperature. The mean temperature was 36.84 °C (standard deviation = 0.61) with extremes of 35.90 and 39.80 °C. More than half of the patients selected for this study were asymptomatic 64.4% (38/59). Clinical signs, when present, were diverse and varied and represented mainly by headache (20.3%) and abdominal pain 13.6% (8/59) (Table 2).

**Table 2:** Clinical characteristics of the study population (non-falciparum cases)

	Number (n = 59)	Percentage
Body temperature (°C)		
35–36.9	40	67.8
37–37.4	12	20.3
≥ 37.5	7	11.9
Clinical status		
Asymptomatic	38	64.4
Symptomatic	21	35.6
Clinical signs		
Clinical signs	12	20.3
Headache	5	8.5
Pale conjunctivitis	8	13.6
Abdominal pain	1	1.7
Arthralgia	4	6.8
Nausea	2	3.2
Vomiting	3	5.1
Diarrhoea	1	1.7

### Prevalence

The overall prevalence of malaria infection was 72.5% (261/360). *Plasmodium falciparum* infection constitute 77. 4% (201/261) and non-falciparum infection constitutes 22.6% (59/261) of all malaria infections. 72.9% (43/59), 15.3% (9/59) and 11.9% (7/59) of the non-falciparum were *P. malariae*, *P. ovale* & *P. malariae* / *P. ovale* mixed infections, respectively. Of the *P. malariae* infections, 90.7% (39/43) were coinfected with *P. falciparum*; 100% (9/9) of the *P. ovale* infections were coinfected with *P. falciparum* 85.7% (6/7) of *P. malariae* & *P.ovale* mixed infection were triple infection with *P. falciparum* (Table 3). No cases of *P. vivax* infection were found.

**Table 3:** Distribution of plasmodial species (non-falciparum cases)

Parasite species	Number (n)	Percentage
*Plasmodium falciparum* + *P. malariae*	39	66.1
*P. falciparum* + *P. malariae* + *P. ovale*	6	10.2
*P. falciparum* + *P. ovale*	9	15.2
*P. malariae*	4	6.8
*P. malariae* + *P. ovale*	1	1.7
Total	59	100.0

The *Plasmodium* indices were 77.6% and 72.3% in the rainy and dry seasons, respectively. Depending on locality, the *Plasmodium* index was 59.6% (53/89) in Grand-Bassam, 79.8% (103/129) in Abengourou, and 81% (115/142) in San Pedro. In most cases, co-infection with *P. falciparum* was observed, and these cases mainly involved asymptomatic malaria regardless of the locality, while only 6.8% (4/59) of the *P. malariae* cases were monoinfected.

*Plasmodium malariae* was the most frequently encountered species in 84.7% (49/59) of the cases of co-infection with *P. falciparum* and *P. ovale*, *Plasmodium ovale* accounted for 27.1% (16/59) of cases, including co-infections (Table 4).

**Table 4:** Distribution of cases according to locality and status (non-falciparum cases)

	Abengourou		Grand bassam		San pedro		Total
	Asymptomatic (n)	Symptomatic (n)	Asymptomatic (n)	Symptomatic (n)	Asymptomatic (n)	Symptomatic (n)	
*P. falciparum* + *P. malariae*	10	5	4	2	9	9	39
*P. falciparum* + *P. malariae* + *P. ovale*	4	2	0	0	0	0	6
*P. falciparum* + *P. ovale*	3	1	1	0	2	2	9
*P. malariae*	1	0	0	0	3	0	4
*P. malariae* + *P. ovale*	0	0	0	0	1	0	1
Total	18	8	5	2	15	11	59
	26		7		26		

The typing of *P. ovale* subspecies revealed the presence of two subspecies, with a predominance of *P. o. curtisi* with a prevalence of 81.2% (13/16). Both subspecies were found mainly in asymptomatic and female participants without any statistically significant association.

## Discussion

The sex ratio of the study was 0.84 (27/32), similar to that found in studies performed in the Ivory Coast, which were 0.98 and 0.89 [20, 21]. The average age was 8.9 years with 64.4% (38/59) of participants aged between 6 and 10 years. This could be explained by the fact that this study took place in a school setting, however the number of children aged 0–5 (pre-schoolers) who are generally more affected by malaria [1], was very low. A similar result was observed in a study performed in Burkina Faso, with a mean age of 8.1 years [22].

The study observed high malaria prevalence in the rainy season compared to the dry season. The observations have been reported in all malaria infections. In the rainy seasons, the malaria vector prevalence increases due to increases in the breeding sites. Thus, increasing malaria transmission in the rainy seasons [23]. In a study carried out in Burkina Faso, *P. malariae* and *P. ovale* had higher proportions at the end of the rainy part of the study period [22].

The fever observed in 11.9% of the participants (mean axillary temperature 36.4 °C) could be explained by the fact that this study population was largely asymptomatic in 64.4% (38/59) of cases. These schoolchildren were apparently healthy; however, when present, their symptoms were dominated by headache 20.3% (12/59), abdominal pain 13.6% (8/59) and pale conjunctiva 8.5% (5/59). Similarly, several other studies have also reported various clinical signs at inclusion but in different proportions than those in this study, including chills, headache, myalgia, anorexia, and asthenia [24]. According to the work of Li et al*.* [25] in China, most patients with *P. malariae* and *P. ovale* had fever at the time of examination (> 37.5 °C) and a history of fever of 2–5 days.

In this study, after removing the cases of *P. falciparum* mono-infection, a significantly higher prevalence of *P. malariae* 66.1%(39/59) was observed compared to *P. ovale* 15.2%(9/59). However, both *P. malariae* and *P. ovale* were sometimes involved in co-infection with *P. falciparum*. In addition, a study in the Democratic Republic of Congo reported that most *P. malariae* and *P. ovale* infections were associated with *P. falciparum* and that the prevalence of mono-infections of these species was only 1.0 and 0.6%, respectively [26]. Similar findings were observed in other studies in Tanzania and Senegal [27, 28]. In addition, most of these non-falciparum infections were found in asymptomatic individuals, who were, therefore, less likely to seek medical treatment, demonstrating the importance of accurately identifying each species independently. In addition, the need to actively detect and target asymptomatic carriers of infection may allow interventions to reduce transmission of non-falciparum malaria, as asymptomatic carriage appears to be more common with *P. malariae* and *P. ovale* [29, 30]. In addition, previous studies have shown that the use of molecular methods, such as PCR, considerably facilitates an increase in the sensitivity of detection of *P. malariae* and *P. ovale* [3, 24, 28], which would provide real data on the epidemiology of these species.

Furthermore, typing of *P. ovale* subspecies showed a predominance of *P. o. curtisi* compared to *P. o. wallikeri* (18.8%). This is the first study with epidemiological data of these two *P. ovale* subspecies in Côte d’Ivoire. Similar trends have been observed in Africa, China, and in places where clinical isolates have been reported to be more closely related to *P. o. curtisi* than *P. o. wallikeri* [25, 31, 32]. In contrast, a study in Canada reported a predominance of *P. o. wallikeri* [33]. However, in this study in Canada it was found that imported malaria cases were associated with a higher proportion of *P. o. curtisi*. Moreover, *P. o. curtisi* was mainly implicated in malaria cases reported in travellers returning to France [34], Italy [35], and China [36]. Mixed infections with *P. o. curtisi* and *P. o. wallikeri* have been reported in Bangladesh and Gabon [31]. The predominance of *P. o. curtisi* in this study could be explained by the use of the primer pair used for *P. ovale*, namely rOVA1/rOVA2. Indeed, this primer pair has a limitation of preferentially binding or amplifying the *P. ovale curtisi* [18]. In order to avoid this limitation, the rOVA1WC/ rOVA2WC primer pair, which binds to both *P. o. wallikeri* and *P. o. curtisi* without binding to other human malaria parasites, is an alternative [19].

## Conclusions

*Plasmodium falciparum* remains the most widespread malaria species in Côte d’Ivoire, but *P. malariae* and *P. ovale* are endemic with a low rate. However, no study has reported the presence of the subspecies *Plasmodium ovale*. Microscopy does not often reliably distinguish between *P. falciparum*, *P. malariae,* and *P. ovale* in Côte d’Ivoire, where all three species are frequently found both in mono-infection and more often in co-infection. Misdiagnosis of *P. malariae* as *P. ovale*, and vice versa, is common, which leads to inappropriate treatment or no treatment at all, given that many individuals are asymptomatic carriers of these species. Thus, *P. ovale* and *P. malariae* may remain important causes of morbidity in these areas and constitute reservoirs of the parasite. In addition, *P. ovale*, due to the presence of hypnozoites, may be responsible for distant relapses and even severe disease. Because *P. malariae* and *P. ovale* infections usually have low parasitaemia and occur as mixed infections with *P. falciparum* and *P. vivax*, molecular detection methods provide a useful tool for the diagnosis of the disease. Molecular detection methods provide an accurate estimate of malarial epidemiology.

## Data Availability

Data supporting our study can be provided by demand from the corresponding author (email: sebastienmiezan@yahoo.fr).

## Abbreviations

CRLP: Centre de Recherche et de Lutte contre le Paludisme
NIPH: National Institute of Public Health
PCR: Polymerase chain reaction
